# Association between depression and cardiometabolic multimorbidity: A protocol for a systematic review and meta-analysis

**DOI:** 10.1371/journal.pone.0352464

**Published:** 2026-06-25

**Authors:** Myo Zin Oo, Soe Sandi Tint, Somdeth Bodhisane

**Affiliations:** 1 Research Institute for Health Sciences, Chiang Mai University, Chiang Mai, Thailand; 2 Global Health and Chronic Conditions Research Center, Chiang Mai University, Chiang Mai, Thailand; 3 Department of Family Medicine, Faculty of Medicine, Chiang Mai University, Chiang Mai, Thailand; 4 College of Public Health Sciences, Chulalongkorn University, Bangkok, Thailand; Mansoura University Faculty of Medicine, EGYPT

## Abstract

**Introduction:**

Cardio metabolic multimorbidity (CMM) is increasingly prevalent and associated with substantial morbidity and mortality, yet the role of depression in the clustering of cardiometabolic conditions has not been comprehensively synthesized. This systematic review and meta-analysis aims to synthesize observational evidence on the association between depression and cardiometabolic multimorbidity and to estimate the prevalence of cardiometabolic multimorbidity among adults with depression.

**Methods:**

The protocol was registered in PROSPERO (CRD420251079959) and is reported in accordance with PRISMA-P guidelines. We will include observational studies (cohort, case-control, and cross-sectional) involving adults aged ≥18 years that examine the association between depression and CMM. Depression must be assessed using validated diagnostic criteria, standardized screening instruments, or clinical codes. CMM will be defined as the coexistence of two or more cardiometabolic diseases, including at least one metabolic and one cardiovascular condition. Relevant studies will be identified through systematic searches of PubMed, Embase, and Scopus from inception to September 2026, without language restrictions. Two reviewers will independently screen studies, extract data, and assess risk of bias using the Newcastle-Ottawa Scale (NOS) for cohort and case-control studies and the Joanna Briggs Institute (JBI) Critical Appraisal Checklist for cross-sectional studies. Random-effects meta-analyses will be conducted to pool adjusted association estimates. Where sufficient longitudinal data are available, longitudinal risk estimates will also be synthesized separately. Pooled prevalence of CMM among individuals with depression will also be estimated. Heterogeneity, publication bias, and subgroup analyses according to age, sex, geographic region, depression ascertainment method, and cardiometabolic multimorbidity definition will be conducted where sufficient data are available. Certainty of evidence will be evaluated using the GRADE approach.

**Conclusion:**

This systematic review and meta-analysis is expected to improve the understanding of the relationship between depression and cardiometabolic multimorbidity and to inform future research, clinical practice, and public health strategies.

## Introduction

Cardiometabolic diseases (CMD) are among the leading contributors to global morbidity and mortality and account for a substantial proportion of the worldwide burden attributable to metabolic and cardiovascular risk factors [1]. As populations age and chronic disease risk factors accumulate, multimorbidity has become increasingly common [2,3]. Cardiometabolic multimorbidity (CMM), defined as the coexistence of two or more cardiometabolic conditions [4,5], has emerged as a particularly high-impact clinical phenotype associated with greater healthcare utilization, reduced quality of life, and increased mortality risk [6,7]. Recent evidence further highlights the complex interplay of metabolic, cardiovascular, renal, and microvascular complications across cardiometabolic conditions, underscoring the importance of considering these diseases as interconnected rather than isolated entities [8,9]. At the same time, depression remains one of the most prevalent mental health disorders globally [10] and is associated with increased susceptibility to chronic physical illness [11]. Together, the rising burden of CMM and the widespread prevalence of depression highlight a critical intersection between mental health and physical health with important implications for public health, clinical care, and long-term disease management.

Depression may contribute to cardiometabolic multimorbidity through biological and behavioral pathways, including chronic inflammation [12], autonomic dysregulation [13], adverse health behaviors [14], and associated metabolic abnormalities [15]. Although depression is known to increase the risk of individual cardiometabolic diseases [16], its role in the development of multiple coexisting conditions has received far less attention. Considering multimorbidity is crucial, as the presence of several cardiometabolic conditions leads to more complex clinical needs, greater healthcare use, and substantially increased mortality risk [7]. Examining depression in relation to cardiometabolic multimorbidity may clarify whether it contributes to the clustering of cardiometabolic conditions and could support more integrated approaches to prevention and long-term management.

A substantial body of observational research has examined the association between depression and individual cardiometabolic diseases such as diabetes, coronary heart disease, and hypertension [17,18]. However, far fewer studies have evaluated cardiometabolic multimorbidity as an integrated outcome. The available evidence is fragmented, with considerable variation in study design, measurement of depression, and definitions of cardiometabolic conditions. Although some studies have assessed depression in relation to specific disease combinations, systematic evidence on its association with cardiometabolic multimorbidity remains limited. These gaps highlight the need for a rigorous synthesis of existing findings to better understand whether depression is related to the clustering of cardiometabolic diseases. The present review will focus specifically on cardiometabolic multimorbidity using a predefined operational definition requiring the coexistence of at least one metabolic and one cardiovascular condition. In addition, where data permit, evidence from prospective cohort studies evaluating incident CMM will be distinguished from cross-sectional and case-control studies assessing prevalent associations.

### Objectives

This systematic review and meta-analysis aims to:

1. Quantify the association between depression and cardiometabolic multimorbidity among adults
2. Estimate the pooled prevalence of cardiometabolic multimorbidity among individuals with depression
3. Examine whether the association or prevalence varies according to demographic, methodological, and study characteristics, including age, sex, geographic region, depression ascertainment method, and cardiometabolic multimorbidity definition

## Methods

This systematic review and meta-analysis is registered in PROSPERO (CRD420251079959) and is reported in accordance with the Preferred Reporting Items for Systematic Review and Meta-Analysis Protocols (PRISMA-P) guidelines [19]. The procedures for literature searching, study selection, data extraction, and statistical analysis are informed by methodological guidance from the Cochrane Handbook for Systematic Reviews [20], adapted as needed for observational epidemiological research.

### Eligibility criteria

Studies will be eligible for inclusion if they involve adults aged 18 years or older and report observational data on the association between depression and cardiometabolic multimorbidity (CMM). In this review, CMM will be operationally defined as the coexistence of at least one metabolic condition and at least one cardiovascular condition. Eligible metabolic conditions include type 2 diabetes, dyslipidemia, obesity, metabolic syndrome, and non-alcoholic fatty liver disease (NAFLD), where reported. Eligible cardiovascular conditions include hypertension, coronary heart disease, stroke, heart failure, peripheral artery disease, and atrial fibrillation. Clusters composed solely of metabolic conditions or solely of cardiovascular conditions will not be classified as CMM. This operational definition is intended to ensure consistency in study selection and synthesis while accommodating variation in disease ascertainment across studies.

Depression must be assessed using validated diagnostic methods, including structured clinical interviews based on the Diagnostic and Statistical Manual of Mental Disorders (DSM) or the International Classification of Diseases (ICD), standardized screening instruments such as the Patient Health Questionnaire-9 (PHQ-9) or the Beck Depression Inventory (BDI), or clinical diagnostic codes. Studies reporting current or lifetime depression will be eligible. For studies using screening instruments, depression will be defined according to the threshold specified by the original study. For association analyses, eligible studies must include a clearly defined comparator group of adults without depression, whereas studies including only individuals with depression will be considered for prevalence analyses.

For cohort studies evaluating temporal associations, preference will be given to studies in which depression is assessed at baseline and incident CMM is ascertained during follow-up. Where reported, information on the exclusion of participants with prevalent CMM at baseline will be extracted and considered during evidence synthesis.

We will include cohort, case-control, and cross-sectional studies published as full-text, peer-reviewed articles, with no restriction on language or publication start date. Studies will be excluded if they involve participants younger than 18 years, non-human populations, assess only a single cardiometabolic condition without addressing multimorbidity, define multimorbidity without cardiometabolic components, or measure depression using unvalidated or ambiguous approaches. Interventional studies, reviews, protocols, commentaries, editorials, conference abstracts, and studies lacking sufficient information to ascertain depression status or CMM will also be excluded. No restrictions will be applied regarding geographic region, healthcare setting, or socioeconomic context.

### Information sources

A comprehensive search will be conducted across multiple electronic bibliographic databases, including PubMed, Embase, and Scopus, to identify eligible studies and ensure broad coverage of biomedical, psychiatric, epidemiological, and allied health literature relevant to depression and CMM. Searches will be conducted from database inception to 30 September 2026, with no language restrictions applied. Non-English records identified as potentially eligible will be screened using translated abstracts when available or automated translation tools, and full texts will be translated where necessary for eligibility assessment. To enhance completeness, Google Scholar will also be screened by relevance, and reference lists of included studies and relevant reviews will be manually reviewed. Citation tracking and contact with study authors will be undertaken when clarification or additional information is required.

### Search strategy

A comprehensive search strategy will be developed using a combination of controlled vocabulary terms (e.g., MeSH, Emtree, and database-specific subject headings) and free-text keywords to capture three core concept domains: (1) depression, (2) cardiometabolic diseases, and (3) multimorbidity. Boolean operators (“AND”, “OR”) will be used to combine terms within and across domains, and search strategies will be adapted to the indexing structure of each database listed in the Information Sources section. No language restrictions will be applied at the search stage. The complete PubMed search strategy is provided in S2 File to ensure transparency and reproducibility. A broad search approach will be employed to maximize sensitivity and capture studies using different definitions of depression and cardiometabolic multimorbidity. The predefined operational definition of cardiometabolic multimorbidity used in this review will be applied during study selection and data extraction.

### Study selection

**Fig 1:**
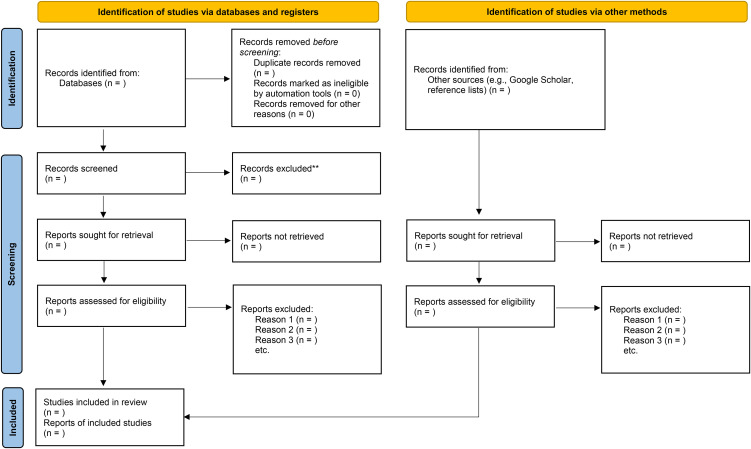
PRISMA flow diagram. Adapted from the PRISMA 2020 statement [21].

All identified records will be imported into EndNote reference management software to facilitate organization and removal of duplicate citations. Following deduplication, titles and abstracts will be screened independently by two reviewers (MZO and SST) against the predefined eligibility criteria. Non-English records will be screened using translated abstracts or translation tools, with full texts translated for eligibility assessment when necessary. Studies judged as potentially relevant by either reviewer will proceed to full-text assessment. The same two reviewers will independently review full-text articles to determine final inclusion. Any disagreements at any stage will be resolved through discussion and consensus; if consensus cannot be reached, a third reviewer (SB) will adjudicate. Reasons for excluding full-text articles will be documented. The study selection process will be reported using a PRISMA 2020 flow diagram template (Fig 1), which will be used to document the number of records identified, screened, assessed for eligibility, and included in the review during the systematic review process [21].

### Data extraction

Data from all eligible studies will be extracted independently by two reviewers (MZO and SST) using a standardized, piloted extraction form, with a third reviewer (SB) resolving disagreements. Where necessary, translated versions of non-English full texts will be used for data extraction. Extracted variables will include:

- **Study characteristics:** author, publication year, country, study design, sample size, and recruitment setting.
- **Participant characteristics:** age, sex distribution, and baseline cardiometabolic status.
- **Exposure and comparator:** method of depression assessment and criteria used to identify non-depressed comparison groups.
- **Outcomes:** each study’s operational definition of CMM, including (a) which cardiometabolic and cardiovascular conditions were included, and (b) how each condition was ascertained. Definitions will be evaluated against the predefined CMM criteria used in this review, which require the coexistence of at least one metabolic condition and one cardiovascular condition, to ensure consistent classification during evidence synthesis. Studies that do not meet the review’s operational CMM definition will be excluded from the primary quantitative synthesis and may be considered in sensitivity analyses or narrative synthesis, as appropriate.
- **Effect estimates:** adjusted association measures (e.g., odds ratios [ORs], relative risks [RRs], hazard ratios [HRs]) and covariates included in adjusted models. Where multiple adjusted estimates are reported, preference will be given to models adjusting for key confounders, including age, sex, socioeconomic status, smoking, body mass index (BMI), and physical activity, where available. For prevalence analyses, the number of participants with depression and those meeting CMM criteria will be extracted.
- **Additional variables:** follow-up duration for cohort studies, funding sources, and reported conflicts of interest.

When required information is unclear or missing, study authors will be contacted. All extracted data will be cross-checked for accuracy before synthesis. When multiple publications are derived from the same cohort or study population, we will assess potential overlap based on study characteristics, recruitment periods, and data sources. Where overlapping populations are identified, priority will be given to the study with the largest sample size, longest follow-up duration (for cohort studies), and most fully adjusted effect estimates. To avoid double-counting participants, only one estimate from an overlapping population will be included in a given meta-analysis. Information from companion publications may be used to supplement missing data where appropriate.

### Outcomes and Prioritization

The primary outcome will be the association between depression and cardiometabolic multimorbidity. For quantitative synthesis, we will extract adjusted effect estimates (e.g., ORs, RRs, HRs) that quantify the association between depression and CMM.

Secondary outcomes will include (1) the pooled prevalence of CMM among individuals with depression and (2) subgroup estimates according to demographic, methodological, and study characteristics, including age, sex, geographic region, depression ascertainment method, and CMM definition. When multiple effect estimates are reported within a study, priority will be given to the most fully adjusted model to reduce confounding. For prevalence analyses, estimates based on clearly defined and validated measures of both depression and CMM will be prioritized.

These outcomes will capture clinically meaningful indicators relevant to understanding the burden and determinants of cardiometabolic multimorbidity among adults with depression.

### Risk of bias and Quality assessment

The methodological quality of all included studies will be assessed independently by two reviewers (MZO and SST), with disagreements resolved through discussion or consultation with a third reviewer (SB). The Newcastle-Ottawa Scale (NOS) will be used to assess the risk of bias of cohort and case-control studies across the domains of selection, comparability, and outcome or exposure assessment. The Joanna Briggs Institute (JBI) Critical Appraisal Checklist for Analytical Cross-Sectional Studies will be used for cross-sectional studies. Risk-of-bias assessments will be conducted independently by two reviewers (MZO and SST), with disagreements resolved through discussion or consultation with a third reviewer (SB). Studies will not be excluded based solely on risk-of-bias ratings; instead, quality assessments will be incorporated into sensitivity analyses and interpretation of the findings.

### Data synthesis

Data synthesis will follow the predefined analytical plan. When studies are sufficiently comparable in population, exposure measurement, and outcome definition, quantitative synthesis will be conducted using random-effects meta-analysis. For association outcomes, adjusted effect estimates will be preferentially extracted and pooled. Where sufficient data are available, ORs, RRs, and HRs will be synthesized separately; if appropriate, effect measures may be converted to a common metric using established methods. Random-effects meta-analysis will be conducted using the restricted maximum likelihood (REML) estimator to generate pooled estimates with 95% confidence intervals. Where sufficient data are available, association estimates will be synthesized separately according to study design. Prospective cohort studies evaluating baseline depression in relation to incident cardiometabolic multimorbidity (CMM) will be analyzed separately from cross-sectional and case-control studies examining prevalent associations. This distinction is intended to account for differences in temporal interpretation across study designs and to avoid combining incident and prevalent outcomes in the same quantitative synthesis. For prevalence outcomes, pooled proportions of CMM among individuals with depression will be calculated using the Freeman-Tukey (FT) double-arcsine transformation, which is commonly used to stabilize variances when pooling proportions, particularly when prevalence estimates are close to 0 or 1. Studies reporting zero events will be retained in the meta-analysis using appropriate variance-stabilizing methods. Where sufficient data are available, subgroup analyses will be conducted according to sampling frame (e.g., population-based, clinical, or high-risk populations), geographic region, and methods used to ascertain depression and CMM to explore potential sources of heterogeneity.

All statistical analyses will be conducted using R software, with meta-analyses performed using established meta-analysis packages. Heterogeneity will be assessed using Cochran’s Q test, the I² statistic, and the between-study variance (τ²). Sources of heterogeneity will be explored through subgroup analyses and meta-regression where sufficient studies are available. Potential moderators may include mean age, sex distribution, geographic region, income setting, healthcare context, depression ascertainment method, and CMM definition. Where sufficient studies are available, evidence will be synthesized separately according to depression ascertainment method (e.g., structured diagnostic interviews, screening instruments, or clinical/administrative codes). Where studies report estimates according to the number of cardiometabolic conditions (e.g., ≥ 2 versus ≥3 conditions), dose–response patterns will be examined narratively or quantitatively where sufficient comparable data are available. Leave-one-out analyses and influence diagnostics will be conducted, where appropriate, to assess the robustness of pooled estimates and identify potential outlying studies. Sensitivity analyses will examine the influence of studies at high risk of bias and the robustness of findings to alternative analytical decisions. Where sufficient studies are available, additional sensitivity analyses will be conducted according to depression ascertainment method (clinical diagnosis versus screening instruments), depression severity where reported, CMM definition, study setting, and level of covariate adjustment, and adjustment for key confounders such as age, sex, socioeconomic status, smoking, BMI, and physical activity. Where sufficient data are available, an additional sensitivity analysis will be conducted excluding CMM definitions driven by hypertension-only cardiovascular combinations to assess the robustness of findings to variation in disease classification. If quantitative synthesis is not feasible due to substantial heterogeneity, insufficient data, or incompatible outcome measures, a narrative synthesis will be undertaken to summarize study characteristics, the direction and magnitude of associations, and relevant contextual factors.

### Meta-bias assessment

Potential meta-biases, including publication bias and selective reporting, will be evaluated. When at least ten studies are available for an association meta-analysis, funnel plot asymmetry will be assessed visually and using Egger’s regression test. Small-study effects will also be explored through sensitivity analyses and comparisons according to study precision. Publication bias assessments will be interpreted cautiously when fewer than ten studies are available and are not expected to be informative for prevalence meta-analyses due to methodological limitations. Where asymmetry is detected, potential explanations such as heterogeneity, methodological limitations, or selective reporting will be examined. Reporting biases will also be considered qualitatively during interpretation of the findings.

### Certainty of evidence

The certainty of evidence for each primary and secondary outcome will be assessed using the GRADE approach, evaluating domains including risk of bias, inconsistency, indirectness, imprecision, and publication bias. Two reviewers (MZO and SST) will independently rate the certainty of evidence, with disagreements resolved by discussion or consultation with a third reviewer (SB). Each outcome will be assigned with an overall certainty rating of high, moderate, low, or very low according to GRADE guidance. Summary of Findings (SoF) tables will be generated to present pooled effect estimates alongside GRADE certainty ratings.

### Ethics and dissemination

As this study is a systematic review of previously published data, ethical approval is not required. No primary data will be collected, and no identifiable information from individual participants will be used. The findings of this review will be disseminated through publication in a peer-reviewed journal. All methodological decisions, including any protocol amendments, will be transparently reported in accordance with PRISMA 2020 guidelines. To enhance transparency and reproducibility, the extracted dataset, data extraction forms, and statistical analysis code used for the meta-analyses will be made publicly available in an appropriate open-access repository upon publication of the final systematic review, subject to journal policies and copyright considerations.

### Timeline

At the time of protocol publication, no stages of the review have been initiated (searching, screening, data extraction, or analysis). The literature search is anticipated to commence following publication of the protocol and will include studies published up to 30 September 2026. Screening, data extraction, risk-of-bias assessment, and data synthesis will subsequently be undertaken. The final systematic review is expected to be completed and submitted for publication thereafter.

## Discussion

Cardiometabolic multimorbidity (CMM) represents a growing global health challenge, and emerging evidence suggests that depression may play an important role in its development and progression. However, existing findings are inconsistent, vary in methodological rigor, and have not yet been synthesized through a systematic review focused specifically on CMM as a defined cluster of cardiometabolic diseases. This systematic review and meta-analysis will address this critical gap by providing the first comprehensive synthesis of observational evidence quantifying the association between depression and CMM, as well as estimating the prevalence of CMM among adults with depression. By consolidating findings from diverse populations and settings, this review may clarify whether depression is associated with cardiometabolic multimorbidity and the clustering of multiple cardiometabolic conditions, with important implications for prevention, clinical practice, and health system planning.

A key strength of this review is its explicit focus on CMM as a multimorbidity construct, extending beyond the single-disease outcomes. Although some observational studies have examined multimorbidity, the clustering of cardiometabolic conditions in relation to depression has not been comprehensively or systematically synthesized. This review will therefore provide novel insights by evaluating CMM as a complex clinical phenotype with implications for disease progression, clinical management, and integrated care models.

The methodological rigor of this protocol further strengthens its potential contribution. We will implement a comprehensive search strategy across multiple biomedical, psychiatric, and interdisciplinary databases; apply prespecified eligibility criteria; extract data using standardized procedures; assess methodological quality using validated tools appropriate for observational study designs. Planned quantitative analyses, including random-effects meta-analysis, subgroup analyses and meta-regression exploring demographic, methodological, and study-level characteristics, and sensitivity analyses, will support robust and interpretable findings. The use of the GRADE approach will enhance transparency by rating the certainty of evidence and presenting conclusions. These steps will ensure that the review adheres to high methodological standards and is informative to clinicians, researchers, and policymakers.

The potential implications of this review extend across multiple sectors. For healthcare professionals involved in mental and physical health care, including physicians, nurses, psychologists, and community health workers, the synthesized evidence may help clarify whether depression is associated with CMM and, if so, could support consideration of more integrated approaches to screening, risk assessment, and care delivery. For public health practitioners and policymakers, the review may provide evidence relevant to planning prevention strategies, allocating resources, and designing care models that address potential interactions between mental and physical health. The findings may also inform tailored prevention strategies and support consideration of CMM risk assessment among individuals with depression where appropriate. For patients and communities, clearer evidence regarding the relationship between depression and cardiometabolic multimorbidity may indirectly inform health literacy efforts and support earlier engagement with preventive services, should the findings indicate such a need.

Despite these strengths, several challenges are anticipated. Heterogeneity is likely due to differences in how depression is measured and how CMM is defined across studies, as well as variation in study design, population characteristics, and covariate adjustment. Although no language restrictions will be applied, the availability and accuracy of translations for some non-English full-text articles may vary. Any limitations arising from translation quality will be documented transparently. To address heterogeneity, we will use a random-effects meta-analysis, conduct subgroup and sensitivity analyses, and explore sources of variability through meta-regression where appropriate. Because observational studies are susceptible to residual confounding and potential reverse causation, findings will be interpreted with caution and contextualized using risk-of-bias assessments. Publication bias may also influence the evidence base; when sufficient studies are available, we will perform formal assessments of small-study effects. Finally, subgroup and prevalence analyses may be constrained by limited reporting or underrepresentation of certain regions, which will be acknowledged in the interpretation of results.

## Conclusion

This systematic review and meta-analysis will provide a comprehensive and rigorous synthesis of the available evidence on the relationship between depression and cardiometabolic multimorbidity. By systematically integrating findings across diverse populations and study designs, the review is expected to clarify how mental health and physical health conditions intersect and to generate evidence that can inform future research priorities, support clinical decision-making, and guide public health planning.

## Supporting information

S1 File PRISMA-P Checklist. (DOCX)

S2 File Planned search strategy for the systematic review. (DOCX)
