# Supplementary material for: Association between depression and cardiometabolic multimorbidity: A protocol for a systematic review and meta-analysis
Source: PLoS One. 2026 Jun 25;21(6):e0352464. doi: 10.1371/journal.pone.0352464 (PMC13298763; doi:10.1371/journal.pone.0352464)
Supplement: S2 File — (DOCX) [file pone.0352464.s002.docx]

**Supplementary Material 1. Planned search strategy for the systematic review**

**Search Concepts and Planned Search Terms**

| **Concept** | **Controlled Vocabulary**  **(e.g., MeSH)** | **Keywords (Free Text)** |
| --- | --- | --- |
| Depression | Depression[Mesh]; Depressive Disorder[Mesh] | depression; depressive disorder*; depressive symptom*; major depression; PHQ-9; Beck Depression Inventory |
| Cardiometabolic diseases | Diabetes Mellitus, Type 2[Mesh]; Hypertension[Mesh]; Coronary Disease[Mesh]; Stroke[Mesh]; Dyslipidemias[Mesh]; Heart Failure[Mesh]; Obesity[Mesh]; Metabolic Syndrome[Mesh]; Fatty Liver[Mesh]; Peripheral Arterial Disease[Mesh]; Atrial Fibrillation[Mesh] | type 2 diabetes; T2DM; hypertension; high blood pressure; coronary heart disease; stroke; dyslipidemia; obesity; metabolic syndrome; fatty liver disease; NAFLD; peripheral artery disease; atrial fibrillation; cardiometabolic; heart failure |
| Multimorbidity | Multimorbidity[Mesh]; Comorbidity[Mesh] | multimorbidity; multimorbid*; multi-morbid*; multiple chronic diseases; comorbidity cluster |

**Planned PubMed Search Strategy**

("Depression"[Mesh] OR depression[tiab] OR "depressive disorder"[tiab] OR "depressive symptoms"[tiab] OR "major depression"[tiab] OR "PHQ-9"[tiab] OR "Beck Depression Inventory"[tiab])

AND

("Multimorbidity"[Mesh] OR multimorbidity[tiab] OR multimorbid*[tiab] OR "multi-morbid*"[tiab] OR "multiple chronic condition*"[tiab] OR comorbidity[tiab] OR "comorbidity cluster"[tiab])

AND

("Diabetes Mellitus, Type 2"[Mesh] OR "Hypertension"[Mesh] OR "Coronary Disease"[Mesh]

OR "Stroke"[Mesh] OR "Dyslipidemias"[Mesh] OR "Heart Failure"[Mesh] OR "Obesity"[Mesh] OR "Metabolic Syndrome"[Mesh] OR "Fatty Liver"[Mesh] OR "Peripheral Arterial Disease"[Mesh] OR "Atrial Fibrillation"[Mesh] OR "type 2 diabetes"[tiab] OR T2DM[tiab] OR hypertension[tiab] OR "coronary heart disease"[tiab] OR "coronary artery disease"[tiab] OR stroke[tiab] OR dyslipidemia[tiab] OR obesity[tiab] OR "metabolic syndrome"[tiab] OR NAFLD[tiab] OR "nonalcoholic fatty liver disease"[tiab] OR "fatty liver disease"[tiab] OR "peripheral artery disease"[tiab] OR "atrial fibrillation"[tiab] OR cardiometabolic[tiab] OR "heart failure"[tiab])

No language or publication-type filters will be applied at the search stage.

**Note:** The final search strategies will be adapted to the indexing structure and search functionality of PubMed, Embase, and Scopus prior to implementation.
