# Supplementary material for: Association between depression and cardiometabolic multimorbidity: A protocol for a systematic review and meta-analysis
Source: PLoS One. 2026 Jun 25;21(6):e0352464. doi: 10.1371/journal.pone.0352464 (PMC13298763; doi:10.1371/journal.pone.0352464)
Supplement: Attachment — Submitted filename: Response to reviewers.docx [file pone.0352464.s004.docx]

3 June 2026

Dear respected Editor,

We would like to express our sincere gratitude for your thorough review of this manuscript. Your constructive and insightful comments have greatly contributed to improving the clarity, rigor, and overall quality of the manuscript. Please find our point-by-point responses below.

**Point-by-point responses to the editor:**

| **Sr.** | **Reviewer** | **Comments and Replies** |
| --- | --- | --- |
| 1 | Editor | Conceptual clarity: Clearly distinguish between association, prevalence, and temporally interpretable risk throughout the manuscript. Revise the title and objectives accordingly if cross-sectional evidence is included. |
|  | Author | Thank you for this important comment. We have revised the manuscript to clearly distinguish between association, prevalence, and temporally interpretable risk throughout the protocol. Specifically, we clarified the objectives, eligibility criteria, outcome definitions, and data synthesis plan to distinguish prospective cohort studies evaluating baseline depression and incident cardiometabolic multimorbidity (CMM) from cross-sectional and case-control studies examining prevalent associations. We have also revised the wording throughout the manuscript to avoid conflating association with causality and to ensure that incidence and prevalence outcomes are interpreted according to the underlying study design. |
| 2 | Editor | Definition of CMM: Provide a consistent and explicit definition of cardiometabolic multimorbidity, including a definitive list of eligible conditions, and clarify how discordant study definitions will be handled analytically. |
|  | Author | Thank you for this comment. We have revised the Eligibility Criteria, Data Extraction, and Data Synthesis sections to provide a consistent and explicit operational definition of cardiometabolic multimorbidity (CMM). The manuscript now includes a definitive list of eligible metabolic and cardiovascular conditions and specifies that CMM requires the coexistence of at least one metabolic condition and one cardiovascular condition. We have also clarified how study-specific CMM definitions will be evaluated against the review definition. Studies that do not meet the predefined operational criteria will be excluded from the primary quantitative synthesis and may be considered in sensitivity analyses or narrative synthesis, as appropriate. |
| 3 | Editor | Depression ascertainment: Specify how different definitions (clinical diagnosis, screening tools, administrative codes) will be handled, and consider prespecified subgroup or sensitivity analyses. |
|  | Author | Thank you for this suggestion. We have revised the Eligibility Criteria and Data Synthesis sections to clarify eligible methods of depression ascertainment, including structured clinical diagnoses, validated screening instruments, and clinical or administrative diagnostic codes. We have also prespecified subgroup and sensitivity analyses according to depression ascertainment method and depression severity, where sufficient data are available, to evaluate the potential influence of exposure classification on pooled estimates and study heterogeneity. |
| 4 | Editor | Risk-of-bias assessment: Clearly define which tools will be used for each study design and how assessments will be applied. |
|  | Author | Thank you for this comment. We have revised the Risk of Bias Assessment section and aligned the Abstract accordingly. The manuscript now explicitly specifies the Newcastle–Ottawa Scale (NOS) for cohort and case-control studies and the Joanna Briggs Institute (JBI) Critical Appraisal Checklist for cross-sectional studies. We have also clarified how assessments will be conducted independently by two reviewers, how disagreements will be resolved, and how risk-of-bias judgments will be incorporated into sensitivity analyses and interpretation of the findings. |
| 5 | Editor | Statistical analysis plan: Strengthen and specify methods for meta-analysis, including handling of different effect measures (OR, RR, HR), choice of models, heterogeneity metrics, and criteria for subgroup analyses and meta-regression. |
|  | Author | Thank you for this comment. We have substantially revised the Data Synthesis and Meta-bias Assessment sections to provide a more detailed and prespecified statistical analysis plan. The manuscript now clarifies the handling of different effect measures (ORs, RRs, and HRs), specifies the use of random-effects meta-analysis with the restricted maximum likelihood (REML) estimator, and details the assessment of heterogeneity using Cochran’s Q, I², and τ². We have also expanded the planned subgroup analyses, meta-regression approaches, influence diagnostics, and sensitivity analyses, and clarified that incident and prevalent outcomes will be synthesized separately where appropriate. |
| 6 | Editor | Prevalence synthesis: Clarify transformation methods, handling of zero cells, and stratification by study setting. |
|  | Author | Thank you for this comment. We have revised the Data Synthesis section to provide additional detail regarding prevalence meta-analysis. Specifically, we now specify the use of the Freeman–Tukey double-arcsine transformation for pooling prevalence estimates, clarify the handling of studies reporting zero events using appropriate variance-stabilizing methods, and outline planned subgroup analyses according to sampling frame and study setting (e.g., population-based versus clinical populations) where sufficient data are available. |
| 7 | Editor | Confounding and sensitivity analyses: Prespecify key confounders and outline planned sensitivity analyses. |
|  | Author | Thank you for this comment. We have revised the Data Extraction and Data Synthesis sections to clarify the handling of confounding and planned sensitivity analyses. The protocol now specifies preferential extraction of maximally adjusted effect estimates and records covariates included in adjusted models. We have also prespecified key confounders of interest, including age, sex, socioeconomic status, smoking, BMI, and physical activity. In addition, we have expanded the sensitivity analyses to evaluate the robustness of findings according to risk of bias, depression ascertainment method, depression severity, CMM definition, study setting, level of covariate adjustment, and alternative disease classification approaches. |
| 8 | Editor | Handling of overlapping data: Describe how duplicate populations or overlapping cohorts will be identified and managed. |
|  | Author | Thank you for this comment. We have revised the Data Extraction section to clarify the identification and management of overlapping populations. The protocol now specifies that study characteristics, recruitment periods, settings, and sample descriptions will be examined to identify potential overlap across publications. Where multiple reports originate from the same cohort or overlapping populations, the most comprehensive publication, the largest sample, or the report with the longest follow-up and most relevant outcome data will be retained for quantitative synthesis to avoid double-counting participants. |
| 9 | Editor | Reporting and consistency: Resolve inconsistencies across sections (e.g., risk-of-bias description, timeline, figures, supplementary materials, and transparency statements). |
|  | Author | Thank you for this comment. We have carefully reviewed the manuscript to improve consistency, transparency, and reporting throughout. Specifically, we aligned the risk-of-bias descriptions across the Abstract and Methods sections, updated the search timeline and review timeline for consistency, completed and updated figure and supplementary material references, and revised the Funding, Acknowledgements, and Data Availability statements to ensure internal consistency and appropriateness for a protocol. We also reviewed the manuscript as a whole to ensure consistency in terminology, definitions, analytical plans, and reporting across all sections. |

With respect and regards,

Authors

Dear respected Reviewer 1,

We would like to express our sincere gratitude for your thorough review of this manuscript. Your constructive and insightful comments have greatly contributed to improving the clarity, rigor, and overall quality of the manuscript. Please find our point-by-point responses below.

**Point-by-point responses to the reviewer 1:**

| **Sr.** | **Reviewer** | **Comments and Replies** |
| --- | --- | --- |
| 1 | Reviewer 1 | “Risk factor” framing in the title implies temporality that cross-sectional and case-control designs cannot establish; plans do not explicitly separate incident CMM analyses from cross-sectional associations. |
|  | Author | Thank you for this valuable comment. We agree that the term “risk factor” may imply temporality that cannot be established by cross-sectional and most case-control studies. Therefore, we revised the title to replace “Risk Factor” with “Association Between.” We also revised the Data Synthesis section to specify that, where sufficient data are available, prospective cohort studies evaluating incident CMM will be synthesized separately from cross-sectional and case-control studies assessing prevalent associations. |
| 2 | Reviewer 1 | The CMM definition is inconsistently operationalized (e.g., allowing cardiovascular-only clusters if a study labels them CMM) and the list of included metabolic/cardiovascular conditions is incomplete or ambiguous (e.g., hypertension classification, dyslipidemia, obesity, metabolic syndrome). |
|  | Author | Thank you for this important comment. We have revised the Eligibility Criteria and Data Extraction sections to provide a clearer and more consistent operational definition of CMM. Specifically, we removed the exception allowing cardiovascular-only clusters, clarified that CMM requires the coexistence of at least one metabolic and one cardiovascular condition, and added a definitive list of eligible metabolic and cardiovascular conditions, including clarification of the classification of hypertension and other relevant cardiometabolic conditions. |
| 3 | Reviewer 1 | No explicit plan to handle overlapping cohorts and duplicate populations across studies. |
|  | Author | Thank you for this comment. We have revised the Data Extraction section to specify procedures for identifying and managing overlapping cohorts and duplicate populations. Where overlapping populations are identified, the most comprehensive or methodologically relevant study will be retained for quantitative synthesis to avoid double-counting participants. |
| 4 | Reviewer 1 | The risk-of-bias tool(s) are not specified; “established tools” is too vague for a protocol. |
|  | Author | Thank you for this comment. We have revised the Risk of Bias and Quality Assessment section to explicitly specify the tools that will be used for each study design. Specifically, the Newcastle-Ottawa Scale (NOS) will be used for cohort and case-control studies, and the Joanna Briggs Institute (JBI) Critical Appraisal Checklist for Analytical Cross-Sectional Studies will be used for cross-sectional studies. |
| 5 | Reviewer 1 | The statistical synthesis plan lacks detail: reconciliation of effect measures (OR, RR, HR), random-effects estimator choice (e.g., REML, DL), heterogeneity metrics (I2, tau2), small-study effects tests (e.g., Egger), and handling of adjusted vs unadjusted estimates. |
|  | Author | Thank you for this comment. We have revised the Data Synthesis and Meta-bias Assessment sections to provide additional details regarding statistical methods. Specifically, we clarified the preferential use of adjusted effect estimates, the handling of ORs, RRs, and HRs, the use of a random-effects model with the REML estimator, assessment of heterogeneity using I², Cochran’s Q, and τ², and the evaluation of small-study effects using funnel plots and Egger’s regression test where appropriate. |
| 6 | Reviewer 1 | For prevalence meta-analysis, no transformation plan (e.g., Freeman–Tukey), treatment of zero cells, or strategy to stratify by sampling frame (population-based vs clinical) is provided. |
|  | Author | Thank you for this comment. We have revised the Data Synthesis section to further clarify the prevalence meta-analysis approach. Specifically, we now explicitly describe the use of the Freeman-Tukey double-arcsine transformation, the handling of zero-event studies using appropriate variance-stabilizing methods, and planned subgroup analyses according to sampling frame (e.g., population-based versus clinical populations). |
| 7 | Reviewer 1 | Confounding control is not prespecified (e.g., minimal adjustment set), and no sensitivity analyses are outlined (e.g., by depression ascertainment, CMM definition, setting, risk of bias). |
|  | Author | Thank you for this comment. We have revised the Data Extraction and Data Synthesis sections to further specify our approach to confounding control and sensitivity analyses. Specifically, we now indicate preference for adjusted estimates accounting for key confounders where available and have added planned sensitivity analyses according to depression ascertainment method, CMM definition, study setting, level of covariate adjustment, and risk of bias. |
| 8 | Reviewer 1 | Temporality for cohort analyses (baseline depression, incident CMM, excluding prevalent CMM) is not clearly prespecified. |
|  | Author | Thank you for this comment. We have revised the Eligibility Criteria section to clarify the temporal framework for cohort studies. Specifically, we now state that preference will be given to studies assessing depression at baseline and incident CMM during follow-up, and that information on the exclusion of participants with prevalent CMM at baseline will be extracted and considered during evidence synthesis. |
| 9 | Reviewer 1 | Minor inconsistencies and placeholders remain (e.g., “please insert figure,” table artifacts, search strategy not included in the supplement within this text). |
|  | Author | Thank you for this observation. We have carefully reviewed the manuscript and removed remaining placeholder text, including the figure insertion note, and checked the tables for formatting consistency. We have also clarified in the Search Strategy section that the complete PubMed search strategy is provided in Supplementary Material 1 to ensure transparency and reproducibility. The detailed search strategy has been submitted as a separate supplementary file in accordance with the journal’s submission requirements. |
| 10 | Reviewer 1 | The planned search end date (March 2026) should be clarified vis-à-vis the protocol timeline. |
|  | Author | Thank you for this comment. We have revised the manuscript to clarify the planned search timeline and ensure consistency with the current status of the review. Specifically, the anticipated search end date has been updated, and corresponding revisions have been made in the Abstract (Methods), Information Sources, and Timeline sections. Corresponding updates will also be made to the PROSPERO registration record. |
| 11 | Reviewer 1 | The background could better situate the protocol relative to existing systematic reviews on depression and multimorbidity, and clarify how this work will differ (e.g., focus on cardiometabolic clusters, stricter CMM definition, incident outcomes). |
|  | Author | Thank you for this comment. We have revised the Introduction to further clarify the scope and contribution of the review. Specifically, we now emphasize the focus on cardiometabolic multimorbidity using a predefined operational definition and clarify the planned distinction between incident and prevalent associations where data permit. |
| 12 | Reviewer 1 | Please predefine: (a) risk-of-bias tools (e.g., ROBINS-I for non-randomized exposures; Newcastle–Ottawa Scale or JBI tools as appropriate), (b) analytic choices for meta-analysis (random-effects estimator, heterogeneity metrics and interpretation thresholds), (c) approach to harmonizing effect measures (e.g., HR approximated as RR when incidence is low, convert OR to RR as feasible or meta-analyze separately by measure), and (d) separate analytical plans for incidence (cohort) vs cross-sectional prevalence/association. |
|  | Author | Thank you for this helpful suggestion. We have revised the Risk of Bias and Quality Assessment, Eligibility Criteria, and Data Synthesis sections to provide additional methodological detail regarding risk-of-bias assessment, effect measure harmonization, meta-analytic methods, and the separate synthesis of incident and prevalent associations. |
| 13 | Reviewer 1 | Given the central role of definitions, provide a definitive list of metabolic (e.g., type 2 diabetes, dyslipidemia, obesity, metabolic syndrome, NAFLD) and cardiovascular (e.g., CHD, stroke, heart failure, atrial fibrillation, PAD, hypertension—if included, justify) conditions eligible for CMM. |
|  | Author | Thank you for this comment. We have revised the Eligibility Criteria section to provide a definitive list of eligible metabolic and cardiovascular conditions for the operational definition of cardiometabolic multimorbidity, including clarification of the classification of hypertension and other relevant cardiometabolic conditions. |
| 14 | Reviewer 1 | Consider a sensitivity analysis excluding hypertension-only combinations, given classification debates. |
|  | Author | Thank you for this valuable suggestion. We have revised the Data Synthesis section to specify that, where sufficient data are available, a sensitivity analysis excluding CMM definitions driven by hypertension-only cardiovascular combinations will be conducted to assess the robustness of the findings. |
| 15 | Reviewer 1 | Clarify inclusion of lifetime vs current depression, clinical diagnosis vs screening positive (with thresholds, e.g., PHQ-9 ≥10), and plan sensitivity analyses by ascertainment method and severity. |
|  | Author | Thank you for this valuable comment. We have revised the Eligibility Criteria section to clarify that studies reporting either current or lifetime depression will be eligible and that screening-based definitions will follow the thresholds specified by the original studies. We have also revised the Data Synthesis section to specify planned sensitivity analyses according to depression ascertainment method and depression severity, where reported. |
| 16 | Reviewer 1 | For association meta-analysis, prespecify: preference for maximally adjusted estimates, handling of heterogeneous covariate sets (e.g., stratify by presence of key confounders: age, sex, SES, smoking, BMI, physical activity, baseline CMD), and strategy for residual confounding assessment (e.g., E-value as optional). |
|  | Author | Thank you for this valuable suggestion. We have revised the Data Extraction and Data Synthesis sections to clarify our preference for maximally adjusted effect estimates and to specify key confounders of interest, including age, sex, socioeconomic status, smoking, BMI, and physical activity. We have also clarified that sensitivity analyses will consider differences in covariate adjustment where sufficient studies are available. |
| 17 | Reviewer 1 | For prevalence meta-analysis, define the statistical transformation (e.g., Freeman–Tukey double arcsine), model (random-effects with appropriate variance estimator), and subgroup/sensitivity analyses by sampling frame (community vs clinical vs high-risk cohorts), geography/HDI, and ascertainment methods for both exposure and outcomes. |
|  | Author | Thank you for this valuable suggestion. We have revised the Data Synthesis section to further clarify the prevalence meta-analysis approach, including the use of the Freeman–Tukey double-arcsine transformation, random-effects meta-analysis with the REML estimator, and planned subgroup analyses according to sampling frame, geographic region, and methods used to ascertain depression and cardiometabolic multimorbidity where sufficient data are available. |
| 18 | Reviewer 1 | Describe plans for small-study effects/publication bias (funnel plot, Egger/Begg tests) for association meta-analyses, noting limitations of these tools for prevalence studies and when k < 10. |
|  | Author | Thank you for this comment. We have revised the Meta-bias Assessment section to further clarify the evaluation of publication bias and small-study effects. Specifically, we now indicate that funnel plots and Egger’s regression test will be applied when at least ten studies are available for association meta-analyses and note the limitations of these approaches when fewer studies are available or for prevalence meta-analyses. |
| 19 | Reviewer 1 | Outline approaches for outlier and influence diagnostics, leave-one-out analyses, and meta-regression (e.g., by mean age, percent female, region, depression ascertainment, CMM definition) if k permits. |
|  | Author | Thank you for this valuable suggestion. We have revised the Data Synthesis section to further clarify planned meta-regression analyses and robustness assessments. Specifically, we now indicate that, where sufficient studies are available, meta-regression may be conducted according to study characteristics such as age, sex distribution, geographic region, depression ascertainment method, and CMM definition. We also specify the use of leave-one-out analyses and influence diagnostics to assess the robustness of pooled estimates. |
| 20 | Reviewer 1 | Address overlapping samples (e.g., UK Biobank used in multiple reports): define a hierarchy to select non-overlapping estimates or combine appropriately. |
|  | Author | Thank you for this important comment. We have revised the Data Extraction section to provide a clearer approach for handling overlapping study populations. Specifically, we now define a hierarchy based on sample size, follow-up duration, and level of covariate adjustment to guide the selection of estimates from overlapping cohorts and avoid double-counting participants. |
| 21 | Reviewer 1 | The related work summaries are largely focused on digital phenotyping and AI-enabled depression detection (e.g., PHQ-based studies, wearable data), which underscores the heterogeneity and potential misclassification in depression ascertainment across studies—a relevant concern for your inclusion and sensitivity analyses. Your plan to include validated tools (PHQ-9, BDI, clinical codes) is appropriate; consider stratifying by clinical diagnosis versus screening tools and, where possible, by severity thresholds. |
|  | Author | Thank you for this valuable suggestion. We agree that variation in depression ascertainment may contribute to heterogeneity across studies. To address this, we have revised the Eligibility Criteria and Data Synthesis sections to clarify eligible methods of depression ascertainment and to specify planned sensitivity analyses according to depression ascertainment method (clinical diagnosis versus screening instruments) and depression severity, where reported. |
| 22 | Reviewer 1 | There is no direct prior systematic review in the provided summaries on depression and CMM specifically, which supports the novelty of your focus on multimorbidity rather than single conditions. Please expand the manuscript’s related work section to explicitly contrast your protocol with prior multimorbidity-focused reviews (if any) and articulate how your stricter CMM definition adds value. |
|  | Author | Thank you for this helpful comment. We have revised the Introduction to further clarify the scope and contribution of the review. Specifically, we now emphasize the focus on cardiometabolic multimorbidity as a distinct outcome, the use of a predefined operational definition of CMM, and the planned distinction between incident and prevalent associations where data permit. |
| 23 | Reviewer 1 | The review could meaningfully inform integrated care models, screening strategies (e.g., prioritizing CMM risk assessment among those with depression), and tailored prevention policies. |
|  | Author | Thank you for this insightful comment. We have revised the Discussion section to further highlight the potential implications of the review for integrated care models, screening and risk assessment strategies, and tailored prevention approaches for individuals with depression. |
| 24 | Reviewer 1 | It also has potential to highlight disparities by age/sex and region; consider adding subgroup analyses by income setting and healthcare context to improve global relevance. |
|  | Author | Thank you for this valuable suggestion. We have revised the Data Synthesis section to indicate that, where sufficient studies are available, subgroup analyses and meta-regression may explore variation according to age, sex, geographic region, income setting, healthcare context, depression ascertainment method, and CMM definition. |
| 25 | Reviewer 1 | Clarify plans for data/code sharing of the extracted dataset and meta-analytic scripts to enhance reproducibility and uptake. |
|  | Author | Thank you for this valuable suggestion. We have revised the Ethics and Dissemination section to clarify our plans for sharing the extracted dataset, data extraction materials, and statistical analysis code in an appropriate open-access repository to enhance transparency and reproducibility. |
| 26 | Reviewer 1 | Which specific risk-of-bias tools will you use for each study design (cohort, case-control, cross-sectional, prevalence-only), and how will ROBINS-I/JBI/NOS decisions be operationalized? |
|  | Author | Thank you for this question. As revised in the Risk of Bias and Quality Assessment section, the Newcastle-Ottawa Scale (NOS) will be used to assess cohort and case-control studies, while the Joanna Briggs Institute (JBI) Critical Appraisal Checklist for Analytical Cross-Sectional Studies will be used for cross-sectional studies, including studies contributing prevalence estimates. Risk-of-bias assessments will be conducted independently by two reviewers, with disagreements resolved through discussion or consultation with a third reviewer. |
| 27 | Reviewer 1 | Will you analyze incident CMM (from cohort studies with baseline depression and no CMM at baseline) separately from cross-sectional associations, and will you adjust the title to avoid causal language (“risk factor”) if substantial cross-sectional evidence predominates? |
|  | Author | Thank you for this question. As revised in the manuscript, prospective cohort studies evaluating baseline depression in relation to incident cardiometabolic multimorbidity (CMM) will be synthesized separately from cross-sectional and case-control studies assessing prevalent associations. In addition, the title has been revised to avoid causal language and better reflect the observational nature of the evidence being synthesized. |
| 28 | Reviewer 1 | How will you harmonize OR, RR, and HR across studies—will you analyze them separately or convert to a common metric? What estimator and heterogeneity metrics (I2, tau2) will you use? |
|  | Author | Thank you for this question. As revised in the Data Synthesis section, ORs, RRs, and HRs will be synthesized separately where sufficient data are available; when appropriate, effect measures may be converted to a common metric using established methods. Random-effects meta-analysis will be conducted using the REML estimator, and heterogeneity will be assessed using Cochran’s Q test, I², and τ². |
| 29 | Reviewer 1 | Please provide a definitive list of cardiometabolic conditions considered “metabolic” versus “cardiovascular,” and clarify whether hypertension will be classified as cardiovascular, metabolic, or handled in sensitivity analyses. Will dyslipidemia, obesity, metabolic syndrome, and NAFLD be included? |
|  | Author | Thank you for this question. As revised in the Eligibility Criteria section, cardiometabolic multimorbidity (CMM) is defined as the coexistence of at least one metabolic condition and at least one cardiovascular condition. Eligible metabolic conditions include type 2 diabetes, dyslipidemia, obesity, metabolic syndrome, and non-alcoholic fatty liver disease (NAFLD), where reported. Eligible cardiovascular conditions include hypertension, coronary heart disease, stroke, heart failure, peripheral artery disease, and atrial fibrillation. Hypertension has been classified as a cardiovascular condition, and we have additionally specified a sensitivity analysis excluding hypertension-only cardiovascular combinations where sufficient data are available. |
| 30 | Reviewer 1 | For prevalence meta-analysis, what transformation (e.g., Freeman–Tukey) and models will you use? How will you handle zero-event studies and stratify by sampling frame (population-based vs clinical)? |
|  | Author | Thank you for this question. As revised in the Data Synthesis section, prevalence estimates will be pooled using the Freeman–Tukey double-arcsine transformation under a random-effects model with the REML estimator. Studies reporting zero events will be retained using appropriate variance-stabilizing methods. Where sufficient data are available, subgroup analyses will be conducted according to sampling frame, including population-based, clinical, or high-risk populations. |
| 31 | Reviewer 1 | What is your minimal confounder set for selecting adjusted estimates (e.g., age, sex, SES, smoking, BMI, physical activity)? Will you conduct sensitivity analyses by adjustment level?  How will you handle overlapping cohorts and duplicate populations across publications (e.g., sample hierarchy rules)? |
|  | Author | Thank you for these questions. As revised in the Data Extraction and Data Synthesis sections, preference will be given to adjusted estimates accounting for key confounders, including age, sex, socioeconomic status, smoking, BMI, and physical activity, where available, and sensitivity analyses will consider level of covariate adjustment where sufficient studies are available. We also clarified that overlapping cohorts will be managed using a predefined hierarchy based on sample size, follow-up duration, and level of covariate adjustment to avoid double-counting participants. |
| 32 | Reviewer 1 | Will you stratify analyses by depression ascertainment (clinical diagnosis vs screening, instrument thresholds, current vs lifetime depression) and by depression severity or duration? |
|  | Author | Thank you for this question. As revised in the Eligibility Criteria section, studies reporting current or lifetime depression will be eligible, and screening-based definitions will follow thresholds specified by the original studies. In the Data Synthesis section, we have specified that sensitivity analyses will be conducted by depression ascertainment method, including clinical diagnosis versus screening instruments, and by depression severity where reported. |
| 33 | Reviewer 1 | Will you examine dose–response relationships (e.g., number of cardiometabolic conditions ≥2 vs ≥3) and conduct meta-regression by mean age, sex distribution, region, and study setting? |
|  | Author | Thank you for this question. As revised in the Data Synthesis section, meta-regression may be conducted where sufficient studies are available using moderators such as mean age, sex distribution, geographic region, income setting, healthcare context, depression ascertainment method, and CMM definition. We have also clarified that dose–response patterns according to the number of cardiometabolic conditions will be examined narratively or quantitatively where sufficient comparable data are available. |
| 34 | Reviewer 1 | Please clarify the planned search end date (March 2026) relative to registration and publication timelines, and whether you plan to update the search prior to final submission. |
|  | Author | Thank you for this question. We have revised the manuscript to clarify the planned search timeline and current status of the review. The anticipated search end date has been updated and corresponding revisions have been made in the Abstract, Information Sources, and Timeline sections. We also intend to update the PROSPERO registration record to ensure consistency with the revised protocol. |

With respect and regards,

Authors

Dear respected Reviewer 2,

We would like to express our sincere gratitude for your thorough review of this manuscript. Your constructive and insightful comments have greatly contributed to improving the clarity, rigor, and overall quality of the manuscript. Please find our point-by-point responses below.

**Point-by-point responses to the reviewer 2:**

| **Sr.** | **Reviewer** | **Comments and Replies** |
| --- | --- | --- |
| 1 | Reviewer 2 | My first concern is conceptual. The title and stated objective frame depression as a “risk factor” for cardiometabolic multimorbidity, yet the eligibility criteria include cross-sectional studies and the secondary outcome is prevalence among individuals with depression. This creates an unresolved tension between causally oriented language and designs that cannot establish temporality. The authors should therefore refine the wording throughout the manuscript so that the protocol clearly distinguishes association, prevalence, and temporally interpretable risk estimates. At present, the manuscript moves too freely between these concepts, which weakens the conceptual discipline of the protocol. |
|  | Author | Thank you for this important comment. We agree that causal language should be used cautiously when synthesizing evidence from multiple observational study designs. Accordingly, we have revised the title and relevant sections of the manuscript to emphasize associations rather than causality. We have also clarified in the Data Synthesis section that prospective cohort studies evaluating incident cardiometabolic multimorbidity will be analyzed separately from cross-sectional and case-control studies assessing prevalent associations, thereby distinguishing temporally interpretable risk estimates from prevalence-based associations. |
| 2 | Reviewer 2 | My second concern is the operational definition of cardiometabolic multimorbidity itself. The manuscript states that CMM will generally require at least one metabolic and one cardiovascular condition, yet it also allows cardiovascular-only clusters when the original study defines them as CMM. That exception substantially dilutes the internal consistency of the review and risks introducing avoidable heterogeneity at the level of outcome definition. |
|  | Author | Thank you for this important comment. We have revised the Eligibility Criteria section to adopt a consistent operational definition of cardiometabolic multimorbidity (CMM) requiring the coexistence of at least one metabolic condition and at least one cardiovascular condition. Studies defining CMM solely on the basis of metabolic-only or cardiovascular-only clusters will no longer meet the review definition, thereby improving consistency and reducing heterogeneity arising from outcome classification. |
| 3 | Reviewer 2 | The same problem appears again in the extraction plan, where study-level definitions will be “compared” with the review definition, but the protocol does not explain how discordant definitions will be handled analytically. This issue is not minor in substance, because the review’s validity depends heavily on whether all included studies are truly measuring the same construct. The authors should clarify the hierarchy of acceptable definitions, the treatment of borderline phenotypes, and whether sensitivity analyses will exclude studies that do not meet the preferred operational standard. |
|  | Author | Thank you for this important comment. We have revised the Data Extraction section to clarify how study-specific CMM definitions will be evaluated against the review’s operational definition. Studies that do not meet the predefined CMM criteria will be excluded from the primary quantitative synthesis and, where appropriate, considered in sensitivity analyses or narrative synthesis. This approach is intended to improve consistency in outcome classification and reduce heterogeneity arising from discordant definitions. |
| 4 | Reviewer 2 | Third, the protocol remains insufficiently precise in how depression will be classified and synthesized. The manuscript combines structured diagnostic criteria, standardized symptom scales, and administrative or clinical codes under a single exposure category. While this may be practical at the search stage, it is too broad for synthesis without a more explicit plan for handling differential exposure validity. A diagnosis based on DSM/ICD interview is not epistemically equivalent to elevated symptom burden on PHQ-9 or BDI, and neither is interchangeable with routine coding data. Without a prespecified strategy for subgrouping or sensitivity analysis by depression ascertainment method, the pooled estimates may become difficult to interpret. The manuscript would benefit from a clearer analytical plan specifying whether diagnostic interviews, screening-defined depression, and code-based depression will be synthesized together or examined separately. |
|  | Author | Thank you for this important comment. We agree that different methods of depression ascertainment may vary in validity and may contribute to heterogeneity across studies. We have therefore revised the Data Synthesis section to clarify that, where sufficient studies are available, evidence will be synthesized separately according to depression ascertainment method (e.g., structured diagnostic interviews, screening instruments, and clinical or administrative codes). We have also specified corresponding sensitivity analyses to assess the robustness of findings across ascertainment approaches. |
| 5 | Reviewer 2 | Fourth, the statistical plan requires greater methodological discipline. The current text proposes pooling adjusted ORs, RRs, and HRs, but does not explain under what assumptions these measures will be combined, whether conversions will be undertaken, or how studies with materially different outcome incidence will be handled. This is particularly important because the review includes mixed observational designs and may combine cross-sectional prevalence-type associations with longitudinal risk estimates. In parallel, the use of the Freeman–Tukey double-arcsine transformation for prevalence pooling should be justified more carefully, as this transformation is not universally preferred and may complicate interpretability. The protocol also mentions meta-regression, subgroup analysis, and publication bias testing, but the decision rules remain underdeveloped. The analysis section should therefore be revised to specify a more rigorous hierarchy of effect measures, transformation rules, conditions for meta-regression, and the exact circumstances under which publication-bias methods will be considered appropriate. |
|  | Author | Thank you for this important comment. We have revised the Data Synthesis and Meta-bias Assessment sections to provide additional methodological detail regarding the handling of ORs, RRs, and HRs, separate synthesis of incident and prevalent associations, the use of the REML estimator, heterogeneity assessment, meta-regression, and publication-bias evaluation. We have also clarified the rationale for using the Freeman–Tukey double-arcsine transformation for prevalence meta-analysis. |
| 6 | Reviewer 2 | Fifth, there are several protocol-reporting and editorial inconsistencies that should not remain in a finalized submission. The abstract states that risk of bias will be assessed using “established tools,” but the main Methods later specify NOS and either adapted NOS or JBI for cross-sectional studies; the abstract should be aligned with the full protocol. More importantly, the manuscript states that no review stages have been initiated, yet the search is said to run from inception to 31 March 2026 while the timeline also indicates that the review will begin in early 2026 and be completed by late 2026. The reporting of dates therefore feels imprecise and potentially confusing. |
|  | Author | Thank you for these observations. We have revised the Abstract to align with the Methods section by explicitly specifying the risk-of-bias tools that will be used for different study designs. We have also updated the search timeline and related dates throughout the manuscript, including the Abstract, Information Sources, and Timeline sections, to clarify the current status of the review and ensure consistency regarding the planned search period and anticipated review activities. |
| 7 | Reviewer 2 | In addition, Figure 1 remains an uncompleted template, and the Supplementary material section still contains placeholder text rather than a finalized citation to the search strategy file. |
|  | Author | Thank you for this observation. We have carefully reviewed the manuscript and addressed these issues. The placeholder figure insertion note has been removed, and Figure 1 has been revised to clarify that it represents the PRISMA 2020 flow diagram template that will be used to document the study selection process during the systematic review. As this manuscript is a protocol and the review has not yet commenced, study selection numbers are not yet available. We have also clarified in the Search Strategy section that the complete PubMed search strategy is provided in Supplementary Material 1 to ensure transparency and reproducibility. The detailed search strategy has been submitted as a separate supplementary file in accordance with the journal’s submission requirements. |
| 8 | Reviewer 2 | Sixth, there is a nontrivial inconsistency in the transparency statements. The manuscript declares that the authors received no funding for the research, yet the acknowledgements state that Chiang Mai University provided invaluable partial support of the study. Those two statements are not obviously compatible and should be reconciled. Likewise, the Data Availability Statement says that no datasets were generated or analyzed during the present study, but then promises that all relevant data will be made fully available without restriction upon study completion. For a protocol, this wording should be made more precise and internally consistent with journal expectations. The authors should also ensure that the ethics, dissemination, and data-sharing language are presented in a form appropriate for a protocol rather than a completed review. |
|  | Author | Thank you for highlighting these inconsistencies. We have revised the Acknowledgements section to clarify the nature of the support provided by Chiang Mai University and ensure consistency with the Funding statement. We have also revised the Data Availability Statement to reflect the protocol nature of the study, clarifying that no datasets have yet been generated or analyzed and specifying the planned availability of review-related materials upon completion of the systematic review and meta-analysis. |
| 9 | Reviewer 2 | The authors may also wish to consider citing a few recent publications that could provide useful contextual support for the broader discussion of cardiometabolic disease complexity, metabolic therapeutics, and multimodal risk stratification. Some studies appear the most appropriate for possible inclusion: 1. PMID: 40005319. DOI: 10.3390/medicina61020202. This study is relevant because it addresses a major cardiometabolic therapeutic class used across chronic disease populations and may help situate the discussion of cardiometabolic disease burden and complexity. 2. PMID: 41038126. DOI: 10.1016/j.jdiacomp.2025.109178. This article may be helpful because it concerns a lipid-modifying intervention tied to metabolic disease and may enrich the manuscript’s framing of cardiometabolic pathways. 3. PMID: 41189017. DOI: 10.1186/s40942-025-00724-y. This study may be considered because it addresses statin-related therapeutic implications within a disease context linked to metabolic and vascular dysregulation. 4. PMID: 40775566. DOI: 10.1007/s12020-025-04374-w. This publication may also be useful because it reflects the broader systemic consequences of diabetes and may support the manuscript’s discussion of cardiometabolic disease clustering and long-term burden. |
|  | Author | Thank you for these helpful suggestions. We reviewed the recommended publications and incorporated relevant citations into the Introduction to further contextualize the complexity and interconnected nature of cardiometabolic diseases. Specifically, we added recent evidence highlighting the interplay of metabolic, cardiovascular, renal, and microvascular complications across chronic cardiometabolic conditions, which further supports the rationale for examining cardiometabolic multimorbidity as a clustered disease construct. |

With respect and regards,

Authors
