# Supplementary material for: Association between depression and cardiometabolic multimorbidity: A protocol for a systematic review and meta-analysis
Source: PLoS One. 2026 Jun 25;21(6):e0352464. doi: 10.1371/journal.pone.0352464 (PMC13298763; doi:10.1371/journal.pone.0352464)
Supplement: Peer review history

# Peer review: Decision Letter 0

**DOI:** 10.1371/journal.pone.0352464.r001

**Authors:** Mohamed Baklola

30 Apr 2026

PONE-D-25-65918

Depression as a Risk Factor for Cardiometabolic Multimorbidity: A Protocol for a Systematic Review and Meta-Analysis

PLOS One

Dear Dr. Oo,

Thank you for submitting your manuscript to PLOS ONE. After careful consideration, we feel that it has merit but does not fully meet PLOS ONE’s publication criteria as it currently stands. Therefore, we invite you to submit a revised version of the manuscript that addresses the points raised during the review process.

Please submit your revised manuscript by Jun 14 2026 11:59PM. If you will need more time than this to complete your revisions, please reply to this message or contact the journal office at plosone@plos.org. When you're ready to submit your revision, log on to https://www.editorialmanager.com/pone/ and select the 'Submissions Needing Revision' folder to locate your manuscript file.

Please include the following items when submitting your revised manuscript:

- A letter that responds to each point raised by the academic editor and reviewer(s). You should upload this letter as a separate file labeled 'Response to Reviewers'.
- A marked-up copy of your manuscript that highlights changes made to the original version. You should upload this as a separate file labeled 'Revised Manuscript with Track Changes'.
- An unmarked version of your revised paper without tracked changes. You should upload this as a separate file labeled 'Manuscript'.

If you would like to make changes to your financial disclosure, please include your updated statement in your cover letter. Guidelines for resubmitting your figure files are available below the reviewer comments at the end of this letter.

If applicable, we recommend that you deposit your laboratory protocols in protocols.io to enhance the reproducibility of your results. Protocols.io assigns your protocol its own identifier (DOI) so that it can be cited independently in the future. For instructions see: https://journals.plos.org/plosone/s/submission-guidelines#loc-laboratory-protocols. Additionally, PLOS ONE offers an option for publishing peer-reviewed Lab Protocol articles, which describe protocols hosted on protocols.io. Read more information on sharing protocols at https://plos.org/protocols?utm_medium=editorial-email&utm_source=authorletters&utm_campaign=protocols.

As the corresponding author, your ORCID iD is verified in the submission system and will appear in the published article. PLOS supports the use of ORCID, and we encourage all coauthors to register for an ORCID iD and use it as well. Please encourage your coauthors to verify their ORCID iD within the submission system before final acceptance, as unverified ORCID iDs will not appear in the published article. *Only*    the individual author can complete the verification step; PLOS staff *cannot*    verify ORCID iDs on behalf of authors.

We look forward to receiving your revised manuscript.

Kind regards,

Mohamed Baklola

Academic Editor

PLOS One

**Journal Requirements:**

1. When submitting your revision, we need you to address these additional requirements.

Please ensure that your manuscript meets PLOS ONE's style requirements, including those for file naming. The PLOS ONE style templates can be found at

https://journals.plos.org/plosone/s/file?id=wjVg/PLOSOne_formatting_sample_main_body.pdf and

https://journals.plos.org/plosone/s/file?id=ba62/PLOSOne_formatting_sample_title_authors_affiliations.pdf

2. Thank you for stating the following in the Acknowledgments Section of your manuscript:

We gratefully acknowledge Chiang Mai University, Thailand, for its invaluable partial support of this study.

We note that you have provided funding information that is not currently declared in your Funding Statement. However, funding information should not appear in the Acknowledgments section or other areas of your manuscript. We will only publish funding information present in the Funding Statement section of the online submission form.

Please remove any funding-related text from the manuscript and let us know how you would like to update your Funding Statement. Currently, your Funding Statement reads as follows:

The author(s) received no specific funding for this work.

Please include your amended statements within your cover letter; we will change the online submission form on your behalf.

3. Please include captions for your Supporting Information files at the end of your manuscript, and update any in-text citations to match accordingly. Please see our Supporting Information guidelines for more information: http://journals.plos.org/plosone/s/supporting-information.

4. If the reviewer comments include a recommendation to cite specific previously published works, please review and evaluate these publications to determine whether they are relevant and should be cited. There is no requirement to cite these works unless the editor has indicated otherwise.

**Additional Editor Comments:**

Thank you for submitting this protocol on depression and cardiometabolic multimorbidity. The topic is important and the proposed review has clear potential; however, major revision is required to ensure methodological rigor, clarity, and reproducibility.

Please address the following key issues:

- Conceptual clarity: Clearly distinguish between association, prevalence, and temporally interpretable risk throughout the manuscript. Revise the title and objectives accordingly if cross-sectional evidence is included.

- Definition of CMM: Provide a consistent and explicit definition of cardiometabolic multimorbidity, including a definitive list of eligible conditions, and clarify how discordant study definitions will be handled analytically.

- Depression ascertainment: Specify how different definitions (clinical diagnosis, screening tools, administrative codes) will be handled, and consider prespecified subgroup or sensitivity analyses.

- Risk-of-bias assessment: Clearly define which tools will be used for each study design and how assessments will be applied.

- Statistical analysis plan: Strengthen and specify methods for meta-analysis, including handling of different effect measures (OR, RR, HR), choice of models, heterogeneity metrics, and criteria for subgroup analyses and meta-regression.

- Prevalence synthesis: Clarify transformation methods, handling of zero cells, and stratification by study setting.

- Confounding and sensitivity analyses: Prespecify key confounders and outline planned sensitivity analyses.

- Handling of overlapping data: Describe how duplicate populations or overlapping cohorts will be identified and managed.

- Reporting and consistency: Resolve inconsistencies across sections (e.g., risk-of-bias description, timeline, figures, supplementary materials, and transparency statements).

[Note: HTML markup is below. Please do not edit.]

Reviewers' comments:

Reviewer's Responses to Questions

**Comments to the Author**

1. Does the manuscript provide a valid rationale for the proposed study, with clearly identified and justified research questions?

The research question outlined is expected to address a valid academic problem or topic and contribute to the base of knowledge in the field.

Reviewer #1: Yes

Reviewer #2: Yes

**********

2. Is the protocol technically sound and planned in a manner that will lead to a meaningful outcome and allow testing the stated hypotheses?

The manuscript should describe the methods in sufficient detail to prevent undisclosed flexibility in the experimental procedure or analysis pipeline, including sufficient outcome-neutral conditions (e.g. necessary controls, absence of floor or ceiling effects) to test the proposed hypotheses and a statistical power analysis where applicable. As there may be aspects of the methodology and analysis which can only be refined once the work is undertaken, authors should outline potential assumptions and explicitly describe what aspects of the proposed analyses, if any, are exploratory.

Reviewer #1: Yes

Reviewer #2: Yes

**********

3. Is the methodology feasible and described in sufficient detail to allow the work to be replicable?

Descriptions of methods and materials in the protocol should be reported in sufficient detail for another researcher to reproduce all experiments and analyses. The protocol should describe the appropriate controls, sample size calculations, and replication needed to ensure that the data are robust and reproducible.

Reviewer #1: Yes

Reviewer #2: Yes

**********

4. Have the authors described where all data underlying the findings will be made available when the study is complete?

The PLOS Data policy requires authors to make all data underlying the findings described in their manuscript fully available without restriction, with rare exception, at the time of publication. The data should be provided as part of the manuscript or its supporting information, or deposited to a public repository. For example, in addition to summary statistics, the data points behind means, medians and variance measures should be available. If there are restrictions on publicly sharing data—e.g. participant privacy or use of data from a third party—those must be specified.

Reviewer #1: Yes

Reviewer #2: Yes

**********

5. Is the manuscript presented in an intelligible fashion and written in standard English?

PLOS ONE does not copyedit accepted manuscripts, so the language in submitted articles must be clear, correct, and unambiguous. Any typographical or grammatical errors should be corrected at revision, so please note any specific errors here.

Reviewer #1: Yes

Reviewer #2: Yes

**********

6. Review Comments to the Author

Please use the space provided to explain your answers to the questions above and, if applicable, provide comments about issues authors must address before this protocol can be accepted for publication. You may also include additional comments for the author, including concerns about research or publication ethics.

You may also provide optional suggestions and comments to authors that they might find helpful in planning their study.

(Please upload your review as an attachment if it exceeds 20,000 characters)

**Reviewer #1:**    This is a timely and relevant protocol that addresses an important intersection between mental health and cardiometabolic multimorbidity. The registration, broad database coverage without language restrictions, and high-level methodological plan are appropriate and commendable. However, for a protocol publication, key methodological specifications need strengthening to ensure transparency and reproducibility: explicit risk-of-bias tools, detailed synthesis methods for effect-size harmonization and prevalence pooling, prespecified confounder sets and sensitivity analyses, a consistent and comprehensive CMM definition, and clear separation of incident risk versus cross-sectional association. I recommend major revisions.

“Risk factor” framing in the title implies temporality that cross-sectional and case-control designs cannot establish; plans do not explicitly separate incident CMM analyses from cross-sectional associations.

The CMM definition is inconsistently operationalized (e.g., allowing cardiovascular-only clusters if a study labels them CMM) and the list of included metabolic/cardiovascular conditions is incomplete or ambiguous (e.g., hypertension classification, dyslipidemia, obesity, metabolic syndrome).

No explicit plan to handle overlapping cohorts and duplicate populations across studies.

The risk-of-bias tool(s) are not specified; “established tools” is too vague for a protocol.

The statistical synthesis plan lacks detail: reconciliation of effect measures (OR, RR, HR), random-effects estimator choice (e.g., REML, DL), heterogeneity metrics (I2, tau2), small-study effects tests (e.g., Egger), and handling of adjusted vs unadjusted estimates.

For prevalence meta-analysis, no transformation plan (e.g., Freeman–Tukey), treatment of zero cells, or strategy to stratify by sampling frame (population-based vs clinical) is provided.

Confounding control is not prespecified (e.g., minimal adjustment set), and no sensitivity analyses are outlined (e.g., by depression ascertainment, CMM definition, setting, risk of bias).

Temporality for cohort analyses (baseline depression, incident CMM, excluding prevalent CMM) is not clearly prespecified.

Minor inconsistencies and placeholders remain (e.g., “please insert figure,” table artifacts, search strategy not included in the supplement within this text).

The planned search end date (March 2026) should be clarified vis-à-vis the protocol timeline.

The background could better situate the protocol relative to existing systematic reviews on depression and multimorbidity, and clarify how this work will differ (e.g., focus on cardiometabolic clusters, stricter CMM definition, incident outcomes).

Overall, the protocol is sound and appropriately structured, but key methodological details require specification to ensure reproducibility and interpretability. Please predefine: (a) risk-of-bias tools (e.g., ROBINS-I for non-randomized exposures; Newcastle–Ottawa Scale or JBI tools as appropriate), (b) analytic choices for meta-analysis (random-effects estimator, heterogeneity metrics and interpretation thresholds), (c) approach to harmonizing effect measures (e.g., HR approximated as RR when incidence is low, convert OR to RR as feasible or meta-analyze separately by measure), and (d) separate analytical plans for incidence (cohort) vs cross-sectional prevalence/association.

Given the central role of definitions, provide a definitive list of metabolic (e.g., type 2 diabetes, dyslipidemia, obesity, metabolic syndrome, NAFLD) and cardiovascular (e.g., CHD, stroke, heart failure, atrial fibrillation, PAD, hypertension—if included, justify) conditions eligible for CMM. Consider a sensitivity analysis excluding hypertension-only combinations, given classification debates.

Clarify inclusion of lifetime vs current depression, clinical diagnosis vs screening positive (with thresholds, e.g., PHQ-9 ≥10), and plan sensitivity analyses by ascertainment method and severity.

For association meta-analysis, prespecify: preference for maximally adjusted estimates, handling of heterogeneous covariate sets (e.g., stratify by presence of key confounders: age, sex, SES, smoking, BMI, physical activity, baseline CMD), and strategy for residual confounding assessment (e.g., E-value as optional).

For prevalence meta-analysis, define the statistical transformation (e.g., Freeman–Tukey double arcsine), model (random-effects with appropriate variance estimator), and subgroup/sensitivity analyses by sampling frame (community vs clinical vs high-risk cohorts), geography/HDI, and ascertainment methods for both exposure and outcomes.

Describe plans for small-study effects/publication bias (funnel plot, Egger/Begg tests) for association meta-analyses, noting limitations of these tools for prevalence studies and when k < 10.

Outline approaches for outlier and influence diagnostics, leave-one-out analyses, and meta-regression (e.g., by mean age, percent female, region, depression ascertainment, CMM definition) if k permits.

Address overlapping samples (e.g., UK Biobank used in multiple reports): define a hierarchy to select non-overlapping estimates or combine appropriately.

The related work summaries are largely focused on digital phenotyping and AI-enabled depression detection (e.g., PHQ-based studies, wearable data), which underscores the heterogeneity and potential misclassification in depression ascertainment across studies—a relevant concern for your inclusion and sensitivity analyses. Your plan to include validated tools (PHQ-9, BDI, clinical codes) is appropriate; consider stratifying by clinical diagnosis versus screening tools and, where possible, by severity thresholds.

There is no direct prior systematic review in the provided summaries on depression and CMM specifically, which supports the novelty of your focus on multimorbidity rather than single conditions. Please expand the manuscript’s related work section to explicitly contrast your protocol with prior multimorbidity-focused reviews (if any) and articulate how your stricter CMM definition adds value.

The review could meaningfully inform integrated care models, screening strategies (e.g., prioritizing CMM risk assessment among those with depression), and tailored prevention policies. It also has potential to highlight disparities by age/sex and region; consider adding subgroup analyses by income setting and healthcare context to improve global relevance.

Clarify plans for data/code sharing of the extracted dataset and meta-analytic scripts to enhance reproducibility and uptake.

Questions for Authors

Which specific risk-of-bias tools will you use for each study design (cohort, case-control, cross-sectional, prevalence-only), and how will ROBINS-I/JBI/NOS decisions be operationalized?

Will you analyze incident CMM (from cohort studies with baseline depression and no CMM at baseline) separately from cross-sectional associations, and will you adjust the title to avoid causal language (“risk factor”) if substantial cross-sectional evidence predominates?

How will you harmonize OR, RR, and HR across studies—will you analyze them separately or convert to a common metric? What estimator and heterogeneity metrics (I2, tau2) will you use?

Please provide a definitive list of cardiometabolic conditions considered “metabolic” versus “cardiovascular,” and clarify whether hypertension will be classified as cardiovascular, metabolic, or handled in sensitivity analyses. Will dyslipidemia, obesity, metabolic syndrome, and NAFLD be included?

For prevalence meta-analysis, what transformation (e.g., Freeman–Tukey) and models will you use? How will you handle zero-event studies and stratify by sampling frame (population-based vs clinical)?

What is your minimal confounder set for selecting adjusted estimates (e.g., age, sex, SES, smoking, BMI, physical activity)? Will you conduct sensitivity analyses by adjustment level?

How will you handle overlapping cohorts and duplicate populations across publications (e.g., sample hierarchy rules)?

Will you stratify analyses by depression ascertainment (clinical diagnosis vs screening, instrument thresholds, current vs lifetime depression) and by depression severity or duration?

Will you examine dose–response relationships (e.g., number of cardiometabolic conditions ≥2 vs ≥3) and conduct meta-regression by mean age, sex distribution, region, and study setting?

Please clarify the planned search end date (March 2026) relative to registration and publication timelines, and whether you plan to update the search prior to final submission.

**Reviewer #2:**    The manuscript addresses a clinically and epidemiologically relevant question, and the topic has sufficient potential interest for publication. However, in its current form, the protocol still requires careful minor revision before it can be considered methodologically sound and editorially ready. My first concern is conceptual. The title and stated objective frame depression as a “risk factor” for cardiometabolic multimorbidity, yet the eligibility criteria include cross-sectional studies and the secondary outcome is prevalence among individuals with depression. This creates an unresolved tension between causally oriented language and designs that cannot establish temporality. The authors should therefore refine the wording throughout the manuscript so that the protocol clearly distinguishes association, prevalence, and temporally interpretable risk estimates. At present, the manuscript moves too freely between these concepts, which weakens the conceptual discipline of the protocol.

My second concern is the operational definition of cardiometabolic multimorbidity itself. The manuscript states that CMM will generally require at least one metabolic and one cardiovascular condition, yet it also allows cardiovascular-only clusters when the original study defines them as CMM. That exception substantially dilutes the internal consistency of the review and risks introducing avoidable heterogeneity at the level of outcome definition. The same problem appears again in the extraction plan, where study-level definitions will be “compared” with the review definition, but the protocol does not explain how discordant definitions will be handled analytically. This issue is not minor in substance, because the review’s validity depends heavily on whether all included studies are truly measuring the same construct. The authors should clarify the hierarchy of acceptable definitions, the treatment of borderline phenotypes, and whether sensitivity analyses will exclude studies that do not meet the preferred operational standard.

Third, the protocol remains insufficiently precise in how depression will be classified and synthesized. The manuscript combines structured diagnostic criteria, standardized symptom scales, and administrative or clinical codes under a single exposure category. While this may be practical at the search stage, it is too broad for synthesis without a more explicit plan for handling differential exposure validity. A diagnosis based on DSM/ICD interview is not epistemically equivalent to elevated symptom burden on PHQ-9 or BDI, and neither is interchangeable with routine coding data. Without a prespecified strategy for subgrouping or sensitivity analysis by depression ascertainment method, the pooled estimates may become difficult to interpret. The manuscript would benefit from a clearer analytical plan specifying whether diagnostic interviews, screening-defined depression, and code-based depression will be synthesized together or examined separately.

Fourth, the statistical plan requires greater methodological discipline. The current text proposes pooling adjusted ORs, RRs, and HRs, but does not explain under what assumptions these measures will be combined, whether conversions will be undertaken, or how studies with materially different outcome incidence will be handled. This is particularly important because the review includes mixed observational designs and may combine cross-sectional prevalence-type associations with longitudinal risk estimates. In parallel, the use of the Freeman–Tukey double-arcsine transformation for prevalence pooling should be justified more carefully, as this transformation is not universally preferred and may complicate interpretability. The protocol also mentions meta-regression, subgroup analysis, and publication bias testing, but the decision rules remain underdeveloped. The analysis section should therefore be revised to specify a more rigorous hierarchy of effect measures, transformation rules, conditions for meta-regression, and the exact circumstances under which publication-bias methods will be considered appropriate.

Fifth, there are several protocol-reporting and editorial inconsistencies that should not remain in a finalized submission. The abstract states that risk of bias will be assessed using “established tools,” but the main Methods later specify NOS and either adapted NOS or JBI for cross-sectional studies; the abstract should be aligned with the full protocol. More importantly, the manuscript states that no review stages have been initiated, yet the search is said to run from inception to 31 March 2026 while the timeline also indicates that the review will begin in early 2026 and be completed by late 2026. The reporting of dates therefore feels imprecise and potentially confusing. In addition, Figure 1 remains an uncompleted template, and the Supplementary material section still contains placeholder text rather than a finalized citation to the search strategy file. These points are editorial, but they materially affect the professionalism and reproducibility of the protocol and should be corrected before acceptance.

Sixth, there is a nontrivial inconsistency in the transparency statements. The manuscript declares that the authors received no funding for the research, yet the acknowledgements state that Chiang Mai University provided invaluable partial support of the study. Those two statements are not obviously compatible and should be reconciled. Likewise, the Data Availability Statement says that no datasets were generated or analyzed during the present study, but then promises that all relevant data will be made fully available without restriction upon study completion. For a protocol, this wording should be made more precise and internally consistent with journal expectations. The authors should also ensure that the ethics, dissemination, and data-sharing language are presented in a form appropriate for a protocol rather than a completed review.

The authors may also wish to consider citing a few recent publications that could provide useful contextual support for the broader discussion of cardiometabolic disease complexity, metabolic therapeutics, and multimodal risk stratification. Some studies appear the most appropriate for possible inclusion: 1. PMID: 40005319. DOI: 10.3390/medicina61020202. This study is relevant because it addresses a major cardiometabolic therapeutic class used across chronic disease populations and may help situate the discussion of cardiometabolic disease burden and complexity. 2. PMID: 41038126. DOI: 10.1016/j.jdiacomp.2025.109178. This article may be helpful because it concerns a lipid-modifying intervention tied to metabolic disease and may enrich the manuscript’s framing of cardiometabolic pathways. 3. PMID: 41189017. DOI: 10.1186/s40942-025-00724-y. This study may be considered because it addresses statin-related therapeutic implications within a disease context linked to metabolic and vascular dysregulation. 4. PMID: 40775566. DOI: 10.1007/s12020-025-04374-w. This publication may also be useful because it reflects the broader systemic consequences of diabetes and may support the manuscript’s discussion of cardiometabolic disease clustering and long-term burden.

Overall, the manuscript has merit and the proposed review may be of interest to readers, but the present version still contains notable deficiencies in conceptual framing, outcome definition, exposure classification, statistical planning, reporting consistency, and transparency statements. I therefore recommend minor revision. The work could become suitable for publication after careful correction of the points above.

**********

7. PLOS authors have the option to publish the peer review history of their article (what does this mean?). If published, this will include your full peer review and any attached files.

If you choose “no”, your identity will remain anonymous but your review may still be made public.

**Do you want your identity to be public for this peer review?**    For information about this choice, including consent withdrawal, please see our Privacy Policy.

Reviewer #1: **Yes:**    Ricardo Ney Cobucci

Reviewer #2: No

**********

[NOTE: If reviewer comments were submitted as an attachment file, they will be attached to this email and accessible via the submission site. Please log into your account, locate the manuscript record, and check for the action link "View Attachments". If this link does not appear, there are no attachment files.]

To ensure your figures meet our technical requirements, please review our figure guidelines: https://journals.plos.org/plosone/s/figures

You may also use PLOS’s free figure tool, NAAS, to help you prepare publication quality figures: https://journals.plos.org/plosone/s/figures#loc-tools-for-figure-preparation.

NAAS will assess whether your figures meet our technical requirements by comparing each figure against our figure specifications.

# Author comment: Author response to Decision Letter 1

**DOI:** 10.1371/journal.pone.0352464.r002

4 Jun 2026

Dear respected Editor,

We would like to express our sincere gratitude for your thorough review of this manuscript. Your constructive and insightful comments have greatly contributed to improving the clarity, rigor, and overall quality of the manuscript. Please find our point-by-point responses below.

Point-by-point responses to the editor:

1 Editor Conceptual clarity: Clearly distinguish between association, prevalence, and temporally interpretable risk throughout the manuscript. Revise the title and objectives accordingly if cross-sectional evidence is included.

Author Thank you for this important comment. We have revised the manuscript to clearly distinguish between association, prevalence, and temporally interpretable risk throughout the protocol. Specifically, we clarified the objectives, eligibility criteria, outcome definitions, and data synthesis plan to distinguish prospective cohort studies evaluating baseline depression and incident cardiometabolic multimorbidity (CMM) from cross-sectional and case-control studies examining prevalent associations. We have also revised the wording throughout the manuscript to avoid conflating association with causality and to ensure that incidence and prevalence outcomes are interpreted according to the underlying study design.

2 Editor Definition of CMM: Provide a consistent and explicit definition of cardiometabolic multimorbidity, including a definitive list of eligible conditions, and clarify how discordant study definitions will be handled analytically.

Author Thank you for this comment. We have revised the Eligibility Criteria, Data Extraction, and Data Synthesis sections to provide a consistent and explicit operational definition of cardiometabolic multimorbidity (CMM). The manuscript now includes a definitive list of eligible metabolic and cardiovascular conditions and specifies that CMM requires the coexistence of at least one metabolic condition and one cardiovascular condition. We have also clarified how study-specific CMM definitions will be evaluated against the review definition. Studies that do not meet the predefined operational criteria will be excluded from the primary quantitative synthesis and may be considered in sensitivity analyses or narrative synthesis, as appropriate.

3 Editor Depression ascertainment: Specify how different definitions (clinical diagnosis, screening tools, administrative codes) will be handled, and consider prespecified subgroup or sensitivity analyses.

Author Thank you for this suggestion. We have revised the Eligibility Criteria and Data Synthesis sections to clarify eligible methods of depression ascertainment, including structured clinical diagnoses, validated screening instruments, and clinical or administrative diagnostic codes. We have also prespecified subgroup and sensitivity analyses according to depression ascertainment method and depression severity, where sufficient data are available, to evaluate the potential influence of exposure classification on pooled estimates and study heterogeneity.

4 Editor Risk-of-bias assessment: Clearly define which tools will be used for each study design and how assessments will be applied.

Author Thank you for this comment. We have revised the Risk of Bias Assessment section and aligned the Abstract accordingly. The manuscript now explicitly specifies the Newcastle–Ottawa Scale (NOS) for cohort and case-control studies and the Joanna Briggs Institute (JBI) Critical Appraisal Checklist for cross-sectional studies. We have also clarified how assessments will be conducted independently by two reviewers, how disagreements will be resolved, and how risk-of-bias judgments will be incorporated into sensitivity analyses and interpretation of the findings.

5 Editor Statistical analysis plan: Strengthen and specify methods for meta-analysis, including handling of different effect measures (OR, RR, HR), choice of models, heterogeneity metrics, and criteria for subgroup analyses and meta-regression.

Author Thank you for this comment. We have substantially revised the Data Synthesis and Meta-bias Assessment sections to provide a more detailed and prespecified statistical analysis plan. The manuscript now clarifies the handling of different effect measures (ORs, RRs, and HRs), specifies the use of random-effects meta-analysis with the restricted maximum likelihood (REML) estimator, and details the assessment of heterogeneity using Cochran’s Q, I², and τ². We have also expanded the planned subgroup analyses, meta-regression approaches, influence diagnostics, and sensitivity analyses, and clarified that incident and prevalent outcomes will be synthesized separately where appropriate.

6 Editor Prevalence synthesis: Clarify transformation methods, handling of zero cells, and stratification by study setting.

Author Thank you for this comment. We have revised the Data Synthesis section to provide additional detail regarding prevalence meta-analysis. Specifically, we now specify the use of the Freeman–Tukey double-arcsine transformation for pooling prevalence estimates, clarify the handling of studies reporting zero events using appropriate variance-stabilizing methods, and outline planned subgroup analyses according to sampling frame and study setting (e.g., population-based versus clinical populations) where sufficient data are available.

7 Editor Confounding and sensitivity analyses: Prespecify key confounders and outline planned sensitivity analyses.

Author Thank you for this comment. We have revised the Data Extraction and Data Synthesis sections to clarify the handling of confounding and planned sensitivity analyses. The protocol now specifies preferential extraction of maximally adjusted effect estimates and records covariates included in adjusted models. We have also prespecified key confounders of interest, including age, sex, socioeconomic status, smoking, BMI, and physical activity. In addition, we have expanded the sensitivity analyses to evaluate the robustness of findings according to risk of bias, depression ascertainment method, depression severity, CMM definition, study setting, level of covariate adjustment, and alternative disease classification approaches.

8 Editor Handling of overlapping data: Describe how duplicate populations or overlapping cohorts will be identified and managed.

Author Thank you for this comment. We have revised the Data Extraction section to clarify the identification and management of overlapping populations. The protocol now specifies that study characteristics, recruitment periods, settings, and sample descriptions will be examined to identify potential overlap across publications. Where multiple reports originate from the same cohort or overlapping populations, the most comprehensive publication, the largest sample, or the report with the longest follow-up and most relevant outcome data will be retained for quantitative synthesis to avoid double-counting participants.

9 Editor Reporting and consistency: Resolve inconsistencies across sections (e.g., risk-of-bias description, timeline, figures, supplementary materials, and transparency statements).

Author Thank you for this comment. We have carefully reviewed the manuscript to improve consistency, transparency, and reporting throughout. Specifically, we aligned the risk-of-bias descriptions across the Abstract and Methods sections, updated the search timeline and review timeline for consistency, completed and updated figure and supplementary material references, and revised the Funding, Acknowledgements, and Data Availability statements to ensure internal consistency and appropriateness for a protocol. We also reviewed the manuscript as a whole to ensure consistency in terminology, definitions, analytical plans, and reporting across all sections.

With respect and regards,

Authors

Dear respected Reviewer 1,

We would like to express our sincere gratitude for your thorough review of this manuscript. Your constructive and insightful comments have greatly contributed to improving the clarity, rigor, and overall quality of the manuscript. Please find our point-by-point responses below.

Point-by-point responses to the reviewer 1:

1 Reviewer 1 “Risk factor” framing in the title implies temporality that cross-sectional and case-control designs cannot establish; plans do not explicitly separate incident CMM analyses from cross-sectional associations.

Author Thank you for this valuable comment. We agree that the term “risk factor” may imply temporality that cannot be established by cross-sectional and most case-control studies. Therefore, we revised the title to replace “Risk Factor” with “Association Between.” We also revised the Data Synthesis section to specify that, where sufficient data are available, prospective cohort studies evaluating incident CMM will be synthesized separately from cross-sectional and case-control studies assessing prevalent associations.

2 Reviewer 1 The CMM definition is inconsistently operationalized (e.g., allowing cardiovascular-only clusters if a study labels them CMM) and the list of included metabolic/cardiovascular conditions is incomplete or ambiguous (e.g., hypertension classification, dyslipidemia, obesity, metabolic syndrome).

Author Thank you for this important comment. We have revised the Eligibility Criteria and Data Extraction sections to provide a clearer and more consistent operational definition of CMM. Specifically, we removed the exception allowing cardiovascular-only clusters, clarified that CMM requires the coexistence of at least one metabolic and one cardiovascular condition, and added a definitive list of eligible metabolic and cardiovascular conditions, including clarification of the classification of hypertension and other relevant cardiometabolic conditions.

3 Reviewer 1 No explicit plan to handle overlapping cohorts and duplicate populations across studies.

Author Thank you for this comment. We have revised the Data Extraction section to specify procedures for identifying and managing overlapping cohorts and duplicate populations. Where overlapping populations are identified, the most comprehensive or methodologically relevant study will be retained for quantitative synthesis to avoid double-counting participants.

4 Reviewer 1 The risk-of-bias tool(s) are not specified; “established tools” is too vague for a protocol.

Author Thank you for this comment. We have revised the Risk of Bias and Quality Assessment section to explicitly specify the tools that will be used for each study design. Specifically, the Newcastle-Ottawa Scale (NOS) will be used for cohort and case-control studies, and the Joanna Briggs Institute (JBI) Critical Appraisal Checklist for Analytical Cross-Sectional Studies will be used for cross-sectional studies.

5 Reviewer 1 The statistical synthesis plan lacks detail: reconciliation of effect measures (OR, RR, HR), random-effects estimator choice (e.g., REML, DL), heterogeneity metrics (I2, tau2), small-study effects tests (e.g., Egger), and handling of adjusted vs unadjusted estimates.

Author Thank you for this comment. We have revised the Data Synthesis and Meta-bias Assessment sections to provide additional details regarding statistical methods. Specifically, we clarified the preferential use of adjusted effect estimates, the handling of ORs, RRs, and HRs, the use of a random-effects model with the REML estimator, assessment of heterogeneity using I², Cochran’s Q, and τ², and the evaluation of small-study effects using funnel plots and Egger’s regression test where appropriate.

6 Reviewer 1 For prevalence meta-analysis, no transformation plan (e.g., Freeman–Tukey), treatment of zero cells, or strategy to stratify by sampling frame (population-based vs clinical) is provided.

Author Thank you for this comment. We have revised the Data Synthesis section to further clarify the prevalence meta-analysis approach. Specifically, we now explicitly describe the use of the Freeman-Tukey double-arcsine transformation, the handling of zero-event studies using appropriate variance-stabilizing methods, and planned subgroup analyses according to sampling frame (e.g., population-based versus clinical populations).

7 Reviewer 1 Confounding control is not prespecified (e.g., minimal adjustment set), and no sensitivity analyses are outlined (e.g., by depression ascertainment, CMM definition, setting, risk of bias).

Author Thank you for this comment. We have revised the Data Extraction and Data Synthesis sections to further specify our approach to confounding control and sensitivity analyses. Specifically, we now indicate preference for adjusted estimates accounting for key confounders where available and have added planned sensitivity analyses according to depression ascertainment method, CMM definition, study setting, level of covariate adjustment, and risk of bias.

8 Reviewer 1 Temporality for cohort analyses (baseline depression, incident CMM, excluding prevalent CMM) is not clearly prespecified.

Author Thank you for this comment. We have revised the Eligibility Criteria section to clarify the temporal framework for cohort studies. Specifically, we now state that preference will be given to studies assessing depression at baseline and incident CMM during follow-up, and that information on the exclusion of participants with prevalent CMM at baseline will be extracted and considered during evidence synthesis.

9 Reviewer 1 Minor inconsistencies and placeholders remain (e.g., “please insert figure,” table artifacts, search strategy not included in the supplement within this text).

Author Thank you for this observation. We have carefully reviewed the manuscript and removed remaining placeholder text, including the figure insertion note, and checked the tables for formatting consistency. We have also clarified in the Search Strategy section that the complete PubMed search strategy is provided in Supplementary Material 1 to ensure transparency and reproducibility. The detailed search strategy has been submitted as a separate supplementary file in accordance with the journal’s submission requirements.

10 Reviewer 1 The planned search end date (March 2026) should be clarified vis-à-vis the protocol timeline.

Author Thank you for this comment. We have revised the manuscript to clarify the planned search timeline and ensure consistency with the current status of the review. Specifically, the anticipated search end date has been updated, and corresponding revisions have been made in the Abstract (Methods), Information Sources, and Timeline sections. Corresponding updates will also be made to the PROSPERO registration record.

11 Reviewer 1 The background could better situate the protocol relative to existing systematic reviews on depression and multimorbidity, and clarify how this work will differ (e.g., focus on cardiometabolic clusters, stricter CMM definition, incident outcomes).

Author Thank you for this comment. We have revised the Introduction to further clarify the scope and contribution of the review. Specifically, we now emphasize the focus on cardiometabolic multimorbidity using a predefined operational definition and clarify the planned distinction between incident and prevalent associations where data permit.

12 Reviewer 1 Please predefine: (a) risk-of-bias tools (e.g., ROBINS-I for non-randomized exposures; Newcastle–Ottawa Scale or JBI tools as appropriate), (b) analytic choices for meta-analysis (random-effects estimator, heterogeneity metrics and interpretation thresholds), (c) approach to harmonizing effect measures (e.g., HR approximated as RR when incidence is low, convert OR to RR as feasible or meta-analyze separately by measure), and (d) separate analytical plans for incidence (cohort) vs cross-sectional prevalence/association.

Author Thank you for this helpful suggestion. We have revised the Risk of Bias and Quality Assessment, Eligibility Criteria, and Data Synthesis sections to provide additional methodological detail regarding risk-of-bias assessment, effect measure harmonization, meta-analytic methods, and the separate synthesis of incident and prevalent associations.

13 Reviewer 1 Given the central role of definitions, provide a definitive list of metabolic (e.g., type 2 diabetes, dyslipidemia, obesity, metabolic s

Attachment Submitted filename: Response to reviewers.docx

# Editor report: Decision Letter 1

**DOI:** 10.1371/journal.pone.0352464.r003

**Authors:** Mohamed Baklola

10 Jun 2026

Association between Depression and Cardiometabolic Multimorbidity: A Protocol for a Systematic Review and Meta-Analysis

PONE-D-25-65918R1

Dear Dr. Oo,

We’re pleased to inform you that your manuscript has been judged scientifically suitable for publication and will be formally accepted for publication once it meets all outstanding technical requirements.

Within one week, you’ll receive an e-mail detailing the required amendments. When these have been addressed, you’ll receive a formal acceptance letter and your manuscript will be scheduled for publication.

An invoice will be generated when your article is formally accepted. Please note, if your institution has a publishing partnership with PLOS and your article meets the relevant criteria, all or part of your publication costs will be covered. Please make sure your user information is up-to-date by logging into Editorial Manager at Editorial Manager® and clicking the ‘Update My Information' link at the top of the page. For questions related to billing, please contact billing support.

If your institution or institutions have a press office, please notify them about your upcoming paper to help maximize its impact. If they’ll be preparing press materials, please inform our press team as soon as possible -- no later than 48 hours after receiving the formal acceptance. Your manuscript will remain under strict press embargo until 2 pm Eastern Time on the date of publication. For more information, please contact onepress@plos.org.

Kind regards,

Mohamed Baklola

Academic Editor

PLOS One

Additional Editor Comments (optional):

Thank you for your careful revision of the manuscript and for addressing the comments raised during peer review. The authors have adequately responded to the reviewers’ concerns, and the revised protocol has been strengthened accordingly.

The objectives are clearly defined, the proposed methodology is appropriate and transparent, and the manuscript is presented in a clear and organized manner. I have no further substantive comments.

I recommend the manuscript for publication in its current form.

Reviewers' comments:

# Editor report: Acceptance letter

**DOI:** 10.1371/journal.pone.0352464.r004

**Authors:** Mohamed Baklola

PONE-D-25-65918R1

PLOS One

Dear Dr. Oo,

I'm pleased to inform you that your manuscript has been deemed suitable for publication in PLOS One. Congratulations! Your manuscript is now being handed over to our production team.

At this stage, our production department will prepare your paper for publication. This includes ensuring the following:

* All references, tables, and figures are properly cited

* All relevant supporting information is included in the manuscript submission,

* There are no issues that prevent the paper from being properly typeset

You will receive further instructions from the production team, including instructions on how to review your proof when it is ready. Please keep in mind that we are working through a large volume of accepted articles, so please give us a few days to review your paper and let you know the next and final steps.

Lastly, if your institution or institutions have a press office, please let them know about your upcoming paper now to help maximize its impact. If they'll be preparing press materials, please inform our press team within the next 48 hours. Your manuscript will remain under strict press embargo until 2 pm Eastern Time on the date of publication. For more information, please contact onepress@plos.org.

You will receive an invoice from PLOS for your publication fee after your manuscript has reached the completed accept phase. If you receive an email requesting payment before acceptance or for any other service, this may be a phishing scheme. Learn how to identify phishing emails and protect your accounts at https://explore.plos.org/phishing.

If we can help with anything else, please email us at customercare@plos.org.

Thank you for submitting your work to PLOS ONE and supporting open access.

Kind regards,

PLOS ONE Editorial Office Staff

on behalf of

Dr. Mohamed Baklola

Academic Editor

PLOS One
